# Scotch Physicians and Surgeons

**Published:** 1888-03-03

**Authors:** C. G. Furley


### SCOTCH PHYSICIANS AND SURGEONS.

By C. G. FURLEY.

(Continued from p. 363.)

### SIR ROBERT CHRISTISON.

CHRISTISON'S instructor in medicine was Gregory, for whose teaching he conceived a deep and lasting reverence. He resented to the end of his life the easy scorn with which practitioners of later times spoke of his master's methods of treatment, and particularly disliked the modern aversion from blood-letting. Christison was a confirmed phlebotomist in his youth, and he protests that the true reason why blood-letting was given up was not a greater degree of enlightenment among medical men, but a change in the character of the diseases for which that remedy had been employed. He certainly has the right to speak with authority, not merely as having the knowledge of more than one generation to base his statement on, but as having during his student course, when he held the responsible and advantageous position of resident physician at the Royal Infirmary, seen much of every kind of fever then known in Britain, especially relapsing fever, the synocha of Hippocrates. As he says, he studied fever for twenty-one months, five months of which were spent in personal experience of its characteristics; and he himself employed blood-letting till 1833, when he became convinced that it no longer served the desired purpose, but not that it had never done so.

His opinions certainly deserve more respectful attention than they have received. "In 1833 I observed a change come over the main character of the fever—its strongly phlogistic type. No such change had occurred in the second great epidemic of 1827-29. In the less wide-spread fevers, however, which ensued for some years afterwards, the rate and force of pulse, heat of skin, and restlessness, became by degrees much less violent, although the nervous prostration was greater; typhus formed a larger proportion of the cases, and presented oftener its severer forms; and the loss of blood, even in pure synocha, could not be sustained as before, so that faintness came on even after only five or six ounces of blood were withdrawn. Accordingly, I altogether gave up blood-letting in fever; nor has it ever been revived since. The simple reason is, that although we have had great epidemics since —in 1842-43, and 1847-48-49—consisting, as in the earlier epidemics, of both typhus and synocha in its relapsing form, there never has been again that high state of inflammatory reaction of the circulation which formerly made the treatment by blood-letting useful and practicable."

Christison gives this explanation of the disuse of blood-letting merely as a justification of the treatment followed by himself and his teachers, and of the reason for its disuse. The evidence he produces is such as, coming from a man of his integrity, cannot be lightly put aside; and we all know that certain diseases, like plants and animals, become extinct, while new ones make their appearance. A case of leprosy, at present a sad curiosity at University College Hospital, is probably the sole survivor in this country of the once numerous colonies of lepers that everywhere existed; and it is matter of common knowledge that nervous diseases of all kinds have become more frequent. Therefore we may accept Christison's explanation of the change in the character of the epidemic fevers he speaks of, and at the same time draw an inference he does not seem to have perceived—that this change was coincident with and probably due to an alteration in the average constitution of man. Great strength, such as can spare some twenty ounces of blood without being the worse for it, is no longer common, being, in consequence of the introduction of mechanical labour-saving appliances, no longer required—it is one of the commonplace truisms of science, that organs and faculties decay with disuse. With this change came the opportunity for a greater development of intellectual faculties, resulting in an enormously increased nervous sensibility. The man of the latter half of this century is a very much more sensitive—dare we say fidgetty and luxurious —subject than his grandfather. He is touched by other influences, higher, subtler, and more ethereal, it may be; but not tending to the fulfilment of the biologists' requirement for success in life—being a good animal. Different from his ancestor in time of health, he is different also in disease; in both cases, and in mind as well as body, the tendency is to "less inflammation and more nervous prostration."

Though himself in most respects a man of what is termed "the old school," Christison was decidedly modern in his views on surgery. Amputations and the like were abhorrent to him. He regarded operations—the delight of so many skilful wielders of the knife, and the ambition of the aspiring student—as the *ultimum remedium* of the surgeon; and looked on the curative surgery which has defrauded the operating table of so many of the victims as a thing whose province must be enlarged more and more with the advance of knowledge.

Like all the medical students of those days, Christison was not unacquainted with the process known as "resurrectioning," though he did not indulge in it with the zest and frolic of many of his companions. It is characteristic of youth to mingle merriment with everything, and even the very terrible fashion in which the students of those days provided for the necessities of the dissecting-room did not preclude a certain grim humour from pervading the stories of their more or less doughty deeds. When, for example, as often happened, two resurrectioning parties heard of the same object for their search, and each sought to outwit the other—reach the grave first, and take possession of it by standing astride of it and sticking a knife in the sod—a claim of right never disputed; or, again, when a party of students successfully frightened away the watchers at a churchyard by disguising themselves in long white night shirts, and masquerading as ghosts. But when the outraged feelings of the relatives of the dead aroused the public conscience, and the authorities no longer winked at these thefts, the students gave up their forays, and "body-snatching," as it was now called, fell into the hands of another and more disreputable class, who sold to the anatomists corpses obtained by means which the doctors, sometimes conveniently blind, forbore to inquire into. This culminated in the notorious crimes of Burke and Hare, a series of murders almost unequalled in history.

The recipient of the bodies procured by these callous monsters was Dr. Knox, a clever lecturer and anatomist, but a man of coarse and selfish temperament. Popular opinion regarded him as an accomplice of the murderers, and gave its advice on the question of punishment in a doggrel couplet—

"Hang Burke, drown Hare,
And burn Knox, of Surgeon Square."

Burke was hanged; Hare, who became King's evidence, led a pariah's life, and ultimately died in one of the remote islands of Shetland. Dr. Knox was, by the law at least, let alone; indeed, he could not be accused of actual participation in a knowledge of the work of his tools; but his carelessness in not inquiring how Burke and Hare came by the bodies they brought with such unfailing regularity to his dissecting-room cannot be looked upon as blameless. The affair ruined his social position, and though for a while his talent and his notoriety kept his class-room full, after a year or two students began to go elsewhere for teaching. He left Edinburgh, and after that fell upon unprosperous days, and died in London in great poverty. One of the last posts he occupied was that of lecturer, demonstrator, or showman to a travelling company of Ojibbeway Indians.

*(To be continued.)*

